# Report of the Committee of the Medical Society of the State of New York on Pharmaceutical Preparations

**Published:** 1860-06

**Authors:** Edward H. Parker, Howard Townsend, Augustus L. Saunders

**Affiliations:** Albany; Albany; Albany


*Report of the Committee of the Medical Society of the State of New York on Pharmaceutical Preparations.*

The committee to whom certain pharmaceutical preparations were referred, beg leave to report, that these preparations were fluid extracts, so called, presented by Messrs. Tilden & Co., and specimens of sugar-coated pills and boluses, presented by Mr. Reichard, agent for the manufacturers, Messrs. Garnier, Lamoureux & Co., of Paris.

The discussion, which arose upon the motion to appoint this committee, seemed to indicate a desire on the part of the Society for a general inquiry into the value of these comparatively new preparations, and this report will, therefore, take a somewhat wider range than a strict construction of the resolution which calls it forth, might warrant. Your committee are, however, quite confident that no serious objection to this course will be raised.

Almost immediately after the adjournment of the Society, a circular was prepared, setting forth the purposes of the committee, and accompanied by a series of questions, to which answers were desired. A copy of the circular is annexed to this report.

The inquiries were divided under four heads, viz.: A, of fluid extracts; B, of solid extracts; C, of active principles; D, of sugar-coated pills; and it will be best for the divisions of this report to follow the same order.

*A, of Fluid Extracts.*

From the answers to the inquiries of your committee, concerning fluid extracts, it appears that this class of preparations are chiefly furnished to the profession in this State, by Tilden & Co., and by Thayer & Co., but it is not quite clear to which the preference is given. Some speak very decidedly in favor of Thayer's, and assign their reasons very distinctly. The majority, perhaps, of those who appear to have given a thorough trial to both manufacturers, seem to prefer Thayer's, but they are a minority of those who have replied to the inquiries of the committee. Your committee have used both preparations, and there seem to be grounds for preferring Thayer's. Usually the dose is smaller, and the extract has more the appearance of an ordinary tincture. The Tildens' preparations are usually more syrupy in their consistence. On this point your committee would not speak very decidedly, but are quite certain that they ought, in alluding to the subject, to commend a careful comparison of the preparations of both manufacturers to the profession. Such a comparison will probably result in a decided preference being given to certain preparations of one manufacture, and to certain others of the other. A few of the correspondents of the committee speak favorably of the fluid extracts of Keith & Co. These do not appear to be much known to the profession generally. The only one of this manufacture which has been used by your committee, is veratrum viride, and has been so concentrated as to require extreme caution in its use.

As a general rule, practitioners obtain their preparations from druggists, and not directly from the manufacturers. It is noticeable that those who obtain them directly from the manufacturers, speak with more decision of their excellence than others. This may be from their supplies being fresher.

It is very clear that "the strength or value of preparations of the same drug from the same maker," is not constant. Some of the correspondents of your committee complain very much of this inequality; while it is no doubt owing to this fact, that such different opinions are expressed concerning the extracts of the same drug. Thus some correspondents appear to place perfect dependence upon the fluid extract of *veratrum viride*, while others say decidedly that it is entirely worthless. Careful observers find that different samples do differ in their strength; and this fact ought to be distinctly understood. The inevitable variations in the valuable qualities of the plant or other crude drug from which the fluid extracts are prepared, make it imperative on the part of the manufacturer to use every care in the selection of his materials, while the difficulty of detecting these variations makes it doubtful if this difference in the extracts can be entirely avoided. Each new package should, therefore, be carefully tested by the physician when he commences to use it. From this fact it is evident that the fluid extracts of the more active drugs are not so well adapted to the use of the simple prescriber in city practice, as to that of the practitioner who dispenses his own medicines.

The testimony is uniform that in this State the fluid extracts keep very well. In some of the Southern States complaint is made that they ferment.

As to the deposit of sediment, it is not generally noticed to exist in large amount. The chairman of your committee found, in examining thirty-six different extracts of Tildens' manufacture, as they stand in a druggist's shop, that ten of them had no deposit at all; in eight it was slight; in three it was very abundant, sometimes amounting to a quarter of the contents of the bottle, and of a honey-like consistence; and in the remaining preparations the deposit, though not so profuse, was quite marked. Taraxacum seems to be especially apt to deposit a sediment. Professor William Procter, Jr., of Philadelphia, the able editor of the *Pharmaceutical Journal*, in the answers which he kindly made to the inquiries of the committee, says that nearly all of these preparations do deposit a sediment, "but not necessarily to their injury." In such large quantity, however, as some of these specimens gave, it must be injurious.

Comparing fluid extracts with tinctures, some of our correspondents prefer one form, and some the other, the parties being almost equally divided, and both speaking very decidedly. A portion are in doubt. Where they are in favor of using the extracts in preference to the tinctures, the reasons assigned are greater uniformity, smaller dose, less taste, and the smaller quantity of alcohol. Your committee doubt if these advantages are not more imaginary than real, and would place fluid extracts and tinctures *carefully made*, on the same footing. Especially is this true of the preparations of the more potent drugs, as aconite and veratrum viride. The latter is spoken of by the correspondents of your committee, with especial frequency, and has perhaps been most commonly used of all the fluid extracts. As compared with Norwood's tincture, it possesses no advantage as to dose, and in that the portion of alcohol is quite small.

So, too, it is with the tinctures of more common use, as, for instance, of rhubarb. The dose of the extract has to be about, if not quite, as large as that of the tincture; its taste is no pleasanter; its action in no way preferable. In fact, your committee would place them upon precisely the same level with good tinctures, carefully made from the best quality of drugs.

It is curious to observe that there is the most varying estimate of the value of the different extracts, by the different correspondents. One gentleman finds everything made by the Tildens to be perfectly reliable, while others find those made by the same firm to be as entirely unreliable. The truth we suppose to be between these extremes. But there is one fact that may account for a portion of this variation of opinion. Ergot is spoken of most uniformly as inert, or nearly so; the enthusiastic correspondent just quoted, admitting that it is "as variable as any." Cotton root stands in the same class, and is spoken of in the same way. Admitting that the cotton root has the action upon the uterus, which is attributed to it, may it not be a fair conclusion that these extracts have been thought to be inert, because they have been administered when they were not indicated? It is far from being true that ergot will produce uterine contractions under all circumstances, and the inappropriate use of the drug ought not to be allowed to affect our opinion of its potency.

In the hands of your committee, the Fl. ext. of ergot has repeatedly produced excessive nausea, compelling its discontinuance, and a resort to the decoction. The preparation of pink root and senna has been carefully tried, and repeatedly by this committee, and with constant failure. That the diagnosis of the presence of lumbricoides has been correct, was repeatedly proved by the subsequent use of santonine, when these parasites promptly presented themselves in the dejections.

Before leaving this class of preparations, your committee take the liberty to object to the "directions for use," which are attached to the bottles containing these preparations by the Messrs. Tilden. It is true they are put on the back of the bottle, or rather on the side opposite the label containing the name of the preparation. They may not be intended to give directions for popular use, but they have that appearance and tone. Certainly physicians do not need, or ought not to need, such information to be so placed. One of the most, perhaps the most objectionable of those labels, is that upon the cotton root. It reads thus:

" Prepared from the bark of the root. Used as an emmenagogue and parturient. As an emmenagogue it is not inferior to ergot in producing uterine contraction, and more safe. It may be used as a valuable remedy in chlorosis, amenorrhœa, dysmenorrhœa, &c. The cotton root is habitually and effectually resorted to by the Southern slaves for producing abortion, and this without any apparent injury to the general health. Dose, table-spoonful, repeated at discretion."

We are very doubtful of the effects of this drug on the " Southern slaves," but admitting it to be true, such information ought not to be communicated to the public. To produce abortion is, in almost all cases, a criminal action, but one already too common. The assurance that it can be done, without injury to the general health, will add to the frequency of the attempt; and when once the moral sense is blunted by the attempt, if this drug fails, other means will be used. A reliable druggist has informed us that already there is a call for this extract, from persons who can only want it for this purpose, and without the advice of physicians. All the information which the profession requires concerning this preparation, is furnished by the advertisements of this firm, and we trust that they will see the wisdom of removing these explanatory labels.

*B, of Solid Extracts.*

It is the narcotic extracts that are most commonly used, though gentian, nux vomica, dandelion, &c., are occasionally mentioned by the correspondents of your committee. In examining a series of these preparations as found in the shops, one is struck by the similarity of odor which many of them give. It is perhaps best described as a burnt molasses or burnt liquorice odor. The color is very uniformly in such preparations a dark brown or black, rendering it entirely impossible to distinguish one preparation from another if the labels chance to be lost or misplaced. It is not surprising, then, that they should have lost reputation, especially with the older practitioners. The more recent manufacturers, however, have greatly improved them, and we find that a large number of correspondents are quite satisfied with Tilden & Co's preparations. Their conium is especially mentioned with approval. It would, however, be an improvement if these extracts were made more solid, their softness requiring too much inert powder to be added to them to bring them into a sufficiently solid condition to remain in the form of pills, for which they are chiefly used.

A few of the correspondents of your committee speak very highly of the extracts manufactured by Allen & Co., of England. From the examination made of them by your committee, we are quite confident that these may rank first among those now found in market, and we would call the attention of the Society to them. By using care in the selection of the extract, and *great* care is necessary, and keeping it carefully in glass-ware, good extracts of the solid form will keep a long time. Otherwise, and especially if kept in a damp place, they will mould. The preparations of an inferior quality are pretty sure to mould anywhere, unless they have been boiled, and reduced, till none of the original elements remain unchanged.

As there were some reasons for supposing that the use of Cannabis Indica might be increasing among the people, for the sake of its intoxicating effects, your committee ventured to step so far out of the line of their duties as to inquire concerning this. With a single exception, the answers have been that there is no indication of such use. This fact is one on which we may congratulate ourselves as physicians, for its use could scarce fail to lead to a resort to opium, which already is indulged in to a fearful extent.

*C, of Active Principles.*

The preparations to which this name is given do not appear to be much used by the profession. Podophyllin is the only one that is spoken of with any frequency. Of this the most various opinions are expressed, some of the correspondents of your committee condemning it as irregular and violent in its action, while others like this and the other similar preparations so much as to use them largely in the place of extracts.

The preparations from different manufacturers vary extremely in their qualities. The color differs, and the effects when administered differ; and no doubt this is one ground, perhaps the main ground, for the different opinions. It is very doubtful if these preparations can be ranked in the same class as quinia, as being the "active principles" of the plants they represent. It is not so precise a process to obtain podophyllin from the mandrake as it is to obtain morphia from opium, and till that certainty in the manufacture can be attained, their use must be attended by some difficulties. One correspondent says it is "a great pity that there is not a uniform standard formula for their manufacture by pharmaceutists." Such a standard would, perhaps, tend to do away with much of the variation in the strength of different preparations, but would not make them fixed substances like the sulphate of quinia and similar salts. From the answers made to the inquiries of your committee, it would seem that these preparations have not yet attained an established position in the materia medica.

Before leaving this topic, it may be remarked that there are a few of our correspondents who speak of podophyllin as a substitute for calomel. It is more correctly estimated by others as a substitute for scammony or for jalap. Careful observation will show that it resembles calomel in its action but very little, and that resemblance is in its most unimportant peculiarities. It will sometimes produce a tenderness of the gums, but different entirely from the specific effect of mercury upon them; and when the specific effect of mercury is necessary, it would be impossible to attain it by the use of podophyllin.

*D, of Sugar-Coated Pills.*

The coating of pills and boluses with sugar, has for its object, of course, the concealment of the taste of the drugs. To this, the only objection that has been urged is, that it is putting on a resemblance to some forms of quackery.

This is, however, an objection more theoretical than practical. To those who have used these preparations, it is quite clear that the coating does not affect the action of the drugs, while it does conceal their taste, and patients do prefer them to pills not coated.

Several American manufacturers are in the habit of making them, the best being furnished by the Tildens. The French pills, made according to the U. S. Pharmacopœia, by Garnier, Lamoureux & Co., of Paris, are thus far the best of all that are found in the market; but our makers are gradually rivaling them. Great care must be used in their manufacture, to select the best materials, to mix them thoroughly, to coat them well, or the reputation of the makers will not be established or retained. This, however, is true of the makers of all such preparations. "*Improvements*" by the manufacturers are not generally acceptable to the profession. The manufacturer's duties are rather mechanical, and the introduction of varied formulas should be left to medical men. On the use of these preparations we cannot do better than to quote the remark of one of the veterans of this Society. In writing to the committee he says: "To save an honorable profession, to minister to the health, and save the lives of men, we should use all the arts of chemistry and pharmacy to render medicine palatable."

In conclusion, your committee beg leave to thank the gentlemen who have replied to their inquiries, and respectfully submit this report.

Edward H. Parker, M.D.,

Howard Townsend, M.D.,

Augustus L. Saunders, M.D.

*Albany, Feb.* 7, 1860.

